# Morphological analysis of myocardial bridging leading to myocardial ischemia: myocardial coronary coupling

**DOI:** 10.3389/fbioe.2025.1559963

**Published:** 2025-04-03

**Authors:** Guanghao Yu, Zhaokai Ming, Dan Qiao, Zhiguo Cheng, Liandi Li, Wei Guo, Xiaoqiang Ye, Wei Ma, Guangxin Chen, Mingming Ren, Jian Xing, Wenchang Tan, Dongliang Zhao

**Affiliations:** ^1^ Medical Image College, Mudanjiang Medical University, Mudanjiang, Heilongjiang, China; ^2^ Medical Imaging Department, Qiqihar First Hospital, Qiqihar, Heilongjiang, China; ^3^ Department of Pathology, Sir Run Run Shaw Hospital, Zhejiang University, Hangzhou, Zhejiang, China; ^4^ Peking University Shenzhen Graduate School, Shenzhen Bay Laboratory, Guangdong, China; ^5^ The Second Affiliated Hospital of Mudanjiang Medical University, Mudanjiang, Heilongjiang, China; ^6^ Department of Cardiovascular Surgery, Peking University Shenzhen Hospital, Shenzhen, Guangdong, China; ^7^ Basic Medical School, Mudanjiang Medical University, Mudanjiang, Heilongjiang, China; ^8^ PKU-HKUST Shenzhen-Hong Kong Institution, Shenzhen, Guangdong, China; ^9^ Department of Mechanics and Engineering Science, College of Engineering, Peking University, Beijing, China

**Keywords:** bridging, myocardial ischemia, morphological analysis, dual source CT, myocardial coronary coupling

## Abstract

**Introduction:**

Myocardial bridge (MB) is a segment of an otherwise extramyocardial blood vessel that traverses the myocardium. This congenital condition typically lacks obvious clinical manifestations during adolescence. However, as individuals age, the accumulated myocardial pressure on the coronary arteries can lead to non-obstructive coronary ischemia, angina pectoris, and even heart failure. Early diagnosis is crucial for assessing the risk of cardiovascular events.

**Methods:**

This study performed a morphological analysis of MB in 75 patients using dual-source Computed Tomographic Angiography (CTA). Through geometric three-dimensional reconstruction, measurements and statistical analyses were conducted on muscle bridge length, depth, length-to-depth ratio, cross-sectional area, and coronary artery curvature.

**Results:**

This study explores the morphological differences among normal individuals, those with superficial MB, and those with deep MB during diastole and systole under varying conditions of myocardial coronary coupling. The study found that the compression degree is greatest in the deep MB group, with the average compression level being approximately 17 times that of normal individuals and about 4.6 times that of patients with superficial MB.

**Discussion:**

The differences in the average cross-sectional area are more significant than those in the minimum cross-sectional area. The depth of the MB is more closely related to the degree of compression, suggesting that clinical intervention and attention should be focused on deep MBs.

## Introduction

Myocardial bridge (MB) are a segment of epicardial coronary artery that is immersed deep within the myocardial wall. Although MB is a congenital physiological abnormality, it leads to obvious abnormalities in coronary blood supply as people age (Hourmozdi and Schimmel, 2024). Myocardial hypertrophy and abnormal contractile function during the aging process will further increase the diameter of MB vessels (Tarantini et al., 2016). In recent years, several studies have demonstrated a direct relationship between MB and non-obstructive coronary artery myocardial infarction (MINOCA), identifying MB as one independent predictor of MINOCA (Brolin et al., 2015; Montone et al., 2021; Abe et al., 2022; Matta et al., 2022; Rinaldi et al., 2023). The incidence of MB in patients with MINOCA is 3.6 times higher, highlighting the necessity of screening for MB in patients with non-ST-segment elevation myocardial infarction (Matta et al., 2022). Additionally, patients with MB in the context of MINOCA exhibit more severe clinical symptoms, with higher hospitalization rates for angina and increased incidence of adverse cardiac events (ACEs) during long-term follow-up (Montone et al., 2021). This suggests that MB is a potential mechanism for poor outcomes in MINOCA (Matta et al., 2022). The presence of MB can directly contribute to angina. Furthermore, MB can also cause atypical chest pain (Duymun and Misodi, 2020), fatal arrhythmias (Grassi et al., 2021), and may even be a cause of death (Grassi et al., 2020). MBs are mostly involved in the left anterior descending branch, and the incidence varies according to different imaging methods and methods of use (Rovera et al., 2023). The prevalence of the autopsy ranged from 5% to 86%, with the largest autopsy report including 1,056 subjects and a found prevalence of 26%, of which 88% involved left anterior descending (LAD) branch (Risse and Weiler, 1985). The prevalence in one computed tomography-based study was about 22.5% (Donkol and Saad, 2013). Diagnosis of MB through outpatient imaging examination CTA plays an important role in the early detection of myocardial ischemia.

Morphological analysis of MB has consistently been a focal point in clinical research. Parameters such as the length, depth, and degree of systolic compression have been proposed to assess the severity of MB (Claudino Dos Santos et al., 2022). A study involving 109 patients, which utilized both coronary Computed Tomographic Angiography (CTA) and Invasive Coronary Angiography (ICA), examined the morphological characteristics of MB. It found that the systolic compression of MB was influenced by the depth of the tunneled segment, rather than its length. This suggests that the degree of systolic compression is a key factor in assessing the impact of MB (Arjmand Shabestari et al., 2016). Santos through the anatomical study of 57 human cadaver hearts and found a significant relationship between the presence of MB and the left ventricular dominant pattern. The incidence of MB was higher at the middle third of the cardiac axis (Claudino Dos Santos et al., 2022). From a morphological perspective, MB can be classified into superficial (1–2 mm of myocardium) and deep (>2 mm of myocardium) bridges (Santucci et al., 2022). The superficial type of MB does not appear to constrict the artery during systole, but deep muscle bridges, due to their association with the left anterior descending coronary artery, could twist the vessel and thus compromise diastolic flow. Park discussed the relationship between MB and fractional flow reserve (Park et al., 2011). In particular, for hypertrophic cardiomyopathy, establishing functional and morphological thresholds for hemodynamically significant MB is crucial for prognosis (Bruce et al., 2023). During cardiac contractions, the myocardium and coronary arteries work in coupling with different morphological characteristics in diastole and systole. The MB experiences compression from the myocardium, influencing coronary hemodynamics, consequently affecting myocardial blood perfusion, thus establishing a detrimental cycle. However, current morphological analyses of MB often overlook the impact of myocardial coronary coupling movements.

The research purpose of this study was to combine dual-source CT and medical image processing software to study the morphology of MB. This study performed a morphological analysis of MB in 75 patients using dual-source CTA. Through geometric three-dimensional reconstruction, measurements and statistical analyses were conducted on muscle bridge length, depth, length-to-depth ratio, cross-sectional area, and coronary artery curvature. The study also discussed the morphological differences among normal individuals, superficial MBs, and deep MBs under varying conditions of myocardial coronary coupling during diastole and systole.

## Materials and methods

### Study design

The clinical baseline characteristics and the CTA imaging study was approved by the Institutional Review Board for the Qiqihar First Hospital and human subjects gave the signed informed consent. 51 patients with isolated MB were diagnosed with dual source CTA, and 24 patients with no abnormalities were diagnosed with dual source CTA. The superficial MB group consisted of 22 patients, and the deep MB group also consisted of 29 patients.

### Dual-source CT coronary angiography

#### Prior to the examination

Subjects were instructed to remove metal items from their chest and perform iodine allergy tests. Necessary explanations were patiently done to relieve potential psychological pressure of subjects; 18G indwelling needle was placed in the anterior cubital vein or other vein vessels of appropriate thickness; In supine position, 4 european basic electrodes were placed on the chest of the subject, and 4 ECG wires of red, yellow, green and black were connected respectively. The heart rate of the subject was observed in real time. In case of serious heart rate problems such as persistent atrial fibrillation, frequent ventricular fibrillation and premature beats, the examination should be stopped and explained to the subject; The breathing training required the subjects to hold their breath for about 8–10 s and to adapt themselves to the instructions several times. The subjects were also required to lie still during the scanning stroke to prevent motion artifacts.

#### Scanning method

**FIGURE 1:**
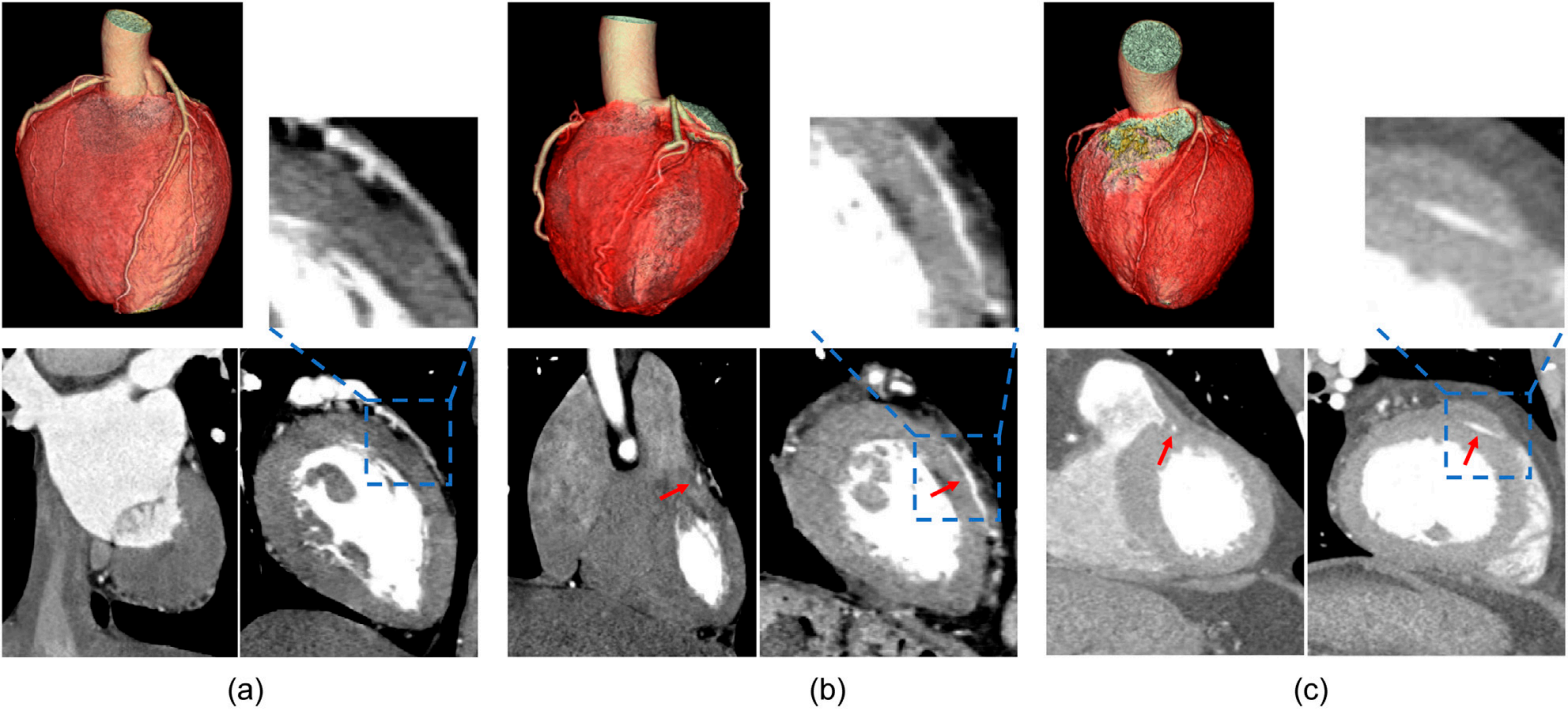
Results of CTA imaging on different cross-sectional planes. **(A)** is the control group, **(B)** is the superficial MB group, **(C)** is the deep MB group.

First, a heart scan similar to chest scan was performed, ranging from about 1 cm below the tracheal bifurcation to about 1 cm below the diaphragmatic surface, and 1–2 cm beyond the heart edge (covering the heart). Tube voltage of 100–120 kV, and a maximum tube current of 380 mAs per rotation. Pitch 0.2–0.5, and automatically match high and low heart rate. Bolus tracking was used. The region of interest (trigger region) was set on the plane of the aortic root, and the trigger threshold of 100 Hounsfield units was set. For all CT examinations, the injection instrument was dual-head power injector (LF Optivantage DH), and the contrast agent was iodoproamine (370mgI/mL). The contrast medium was injected into the bolus injection. And an intravenous test (about 10 mL normal saline) was performed before injection to confirm the absence of subcutaneous leakage. The volume of contrast medium was about 80 mL (adjusted according to body weight), and the injection rate was 4.5–5.5 mL/s. Then 40 mL normal saline was injected at the same rate to flush out and promote the contrast medium in the superior vena cava and right heart system, which was conductive to coronary artery development. After the contrast agent is injected, the automatic tracing starts, and the client can complete the examination with the breath-hold instruction. Patient data is stored in DICOM format. The images of three are shown in Figure 1.

### 3D reconstruction and measurement

All CT images were consensually analyzed by two radiologists familiar with Mimics software (Materialise Inc., Belgium) in the trial team, and the image quality of coronary arteries was semi-quantitatively assessed using a 4-point ranking scale (4, excellent image quality; 3, good image quality; 2, fair image quality; 1, poor image quality). Excellent image quality indicated a clear outline, smooth edges, no motion artifacts, no punctate blur. Image quality was classified as vascular contrast with slight motor artifacts and mild blurring of vascular margins. Fair image quality was defined was moderate motor artifacts and moderate blurred vascularity, no structural discontinuities. Poor image quality was classified as severe motor artifacts, poor contrast of blood vessels, discontinuities. We choose excellent image quality and good image quality to group (Hwang et al., 2010).

**FIGURE 2:**
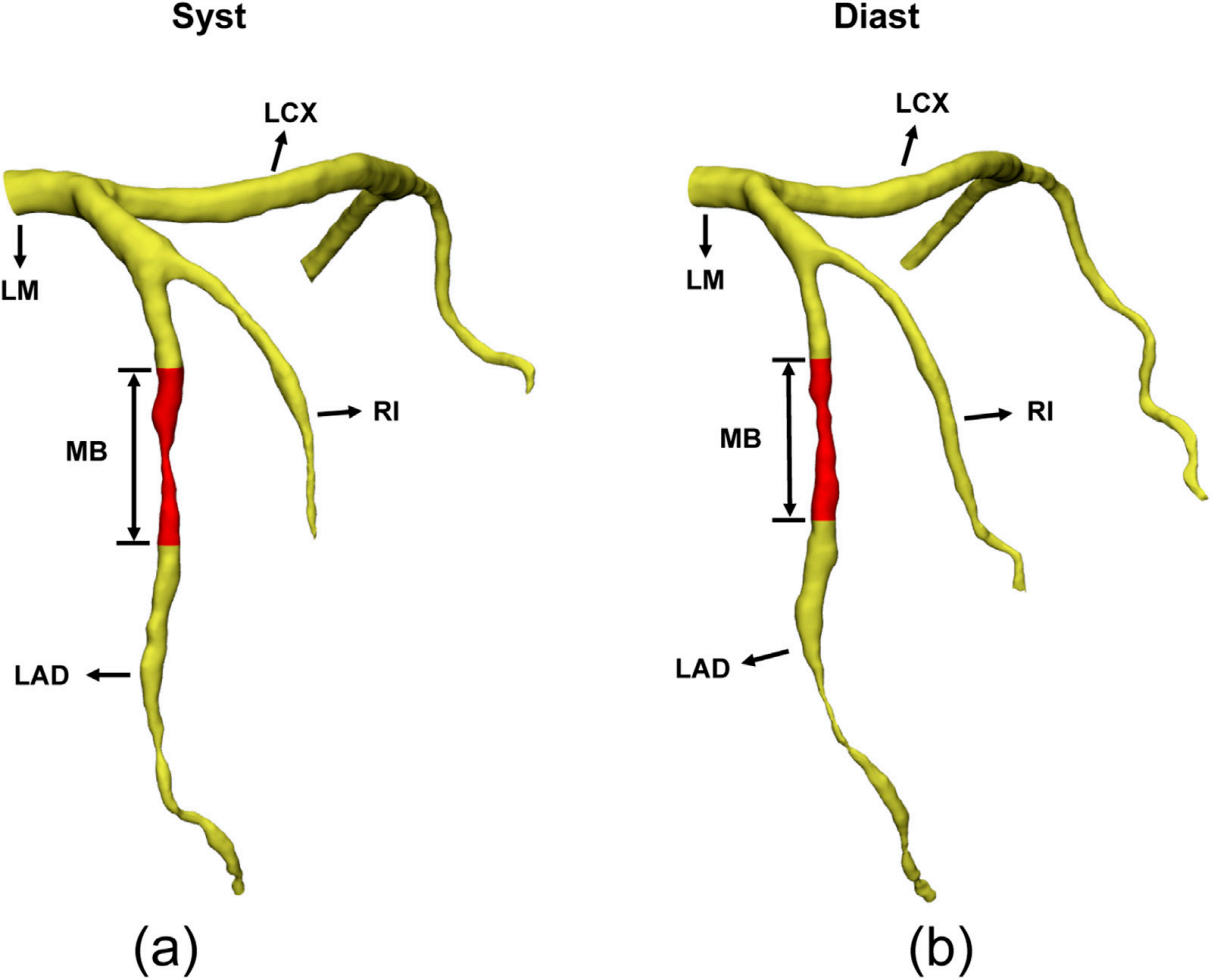
3D reconstruction of MB images in systole **(A)** and diastole **(B)**.

To import CT tomography images into Mimics software, the “Import Images” function was utilized, with the top and bottom of the images manually defined. The software automatically generated sagittal and coronal images from the cross-sectional images. Upon entering the three views, thresholds were used to extract the outlines. The basic principle is to ensure that the selected tissue for reconstruction is highlighted, avoiding contour shading of structures outside the targeted tissue, and adjusting clarity to an appropriate level. The region growing tool in the software was used for threshold-based region growing, and the MB segment was manually reconstructed. After completing the reconstruction, the “Calculate 3D” option was selected from the menu to convert the 2D images into a 3D model. This model was then imported into 3-matic Research 13.0 (Materialise Inc., Belgium), where it was refined using plane cutting and smoothed with the local smoothing function. The 3D reconstructed microstructures were inspected by two seasoned engineers. Finally, the data was exported to Excel after measurements were taken using the software’s built-in centerline measurement tool. The results of the reconstruction and morphological measurements are shown in Figure 2.

### Morphological analysis

The depth of MB: Select the systolic period and diastolic phase respectively, the axial section selects the angle that can observe the thickest part of the myocardial fiber covered on the MB, and measures the vertical distance from the myocardial surface to the lateral wall of the MB to obtain the MB depth.

The length of MB: Measure the coronary centerline length in myocardial bridge segments.

Length depth ratio:

$$Length\;depth\;ratio=\frac{L}{D}\times 100\%$$

*L* means the length of MB, *D* means the deep of the MB.

Minimum Cross-sectional Area: Measure the minimum cross-sectional area of the MB segment during systole and diastole.

Average Cross-sectional Area: Calculate the average cross-sectional area of the MB segment during systole and diastole.

Maximum compression degree:

$$Maximum\;compression\;degree=\frac{{AD}_{\min}-{AS}_{\min}}{{AD}_{\min}}\times 100\%$$

where, ${AD}_{\min}$ is the minimum cross-sectional area during diastole, and ${AS}_{\min}$ is the minimum cross-sectional area during systole.

Average Compression Degree:

$$Average\;compression\;degree=\frac{{AD}_{ave}-{AS}_{ave}}{{AD}_{ave}}\times 100\%$$

where, ${AD}_{ave}$ is the average cross-sectional area during diastole, and ${AS}_{ave}$ is the average cross-sectional area during systole.

Curvature: Measure the maximum, minimum, and average curvature of the MB segment in patients. Based on the centerline of the coronary MB segment, the curvature (K) is calculated using the coordinate equation:

$$K=\frac{\left|y^{\prime \prime }\right|}{{\left(1+y^{\prime 2}\right)}^{\frac{3}{2}}}$$

where, $y^{\prime }$ is the first derivative and $y^{\prime \prime }$ is the second derivative of the centerline function.

Control group morphological measurements: Myocardial bridges were most commonly found in the middle of the left anterior descending branch, which was approximately 14.64 mm in length (Yuan, 2016), and for the purpose of comparison with patients with myocardial bridges, the minimum cross-sectional area, the mean cross-section area and curvature of the control group were obtained from this section of the coronary artery.

### Statistical analysis

SPSS version 26.0 software (SPSS, Chicago, IL, United States) was used for statistical data analyses. Statistical significance was set at P < 0.05. In descriptive statistical analysis, quantitative variables are expressed in mean ± SD, and categorical variables are expressed in frequencies or percentage. Quantitative variables were tested for normal distribution. Comparing the information of age and biochemical indexes (include cholesterol, triglycerides, high-density lipoprotein, low-density lipoprotein and uric acid) between control group and MB patients, the variables conformed to normal distribution, and T-test was used. Comparison of the compositional percentage of gender, smoking and medical history (including hypertension, diabetes, and coronary artery disease) between control group and MB patients was performed using the Chi-Square test. To compare the differences in depth, length and length/depth ratio of muscle bridges in the deep and superficial muscle bridge groups, variables with normal distribution were tested using the t-test, and Mann-Whitney U test was used for non-normal distribution. To compare the differences in cross-sectional area, variations in cross-sectional area during systole and diastole, compression degree and curvature between the deep and superficial muscle bridge groups and the control group, variables with a normal distribution were analyzed using the ANOVA test, *post hoc* multiple comparisons were performed using the Bonferroni test, and non-normally distributed data were analyzed using Kruskal-Wallis tests. The correlations between the length, depth, maximum compression degree, and average compression degree of superficial and deep MB in systole and diastole were analyzed using the Spearman test.

## Results

All clinical baseline information is presented in Table 1. Factors including hypertension, diabetes, coronary heart disease, cholesterol, triglycerides, high-density lipoprotein, low-density lipoprotein, and uric acid levels were compared between the deep MB group and the superficial MB group. In the control group, systolic period was 40.00% ± 4.63% of the cardiac cycle, and the diastolic period was 74.67% ± 3.35% of the cardiac cycle. The systolic period of the superficial MB group and the deep MB group was 37.14% ± 4.58% and 37.09% ± 4.15% of the cardiac cycle, respectively. There was no significant difference between the two groups (p = 0.776).

**TABLE 1:** Clinical characteristics between the MB patients and the control group.

	MB group (n = 51)	Control group (n = 24)	X^2^/t value	p value
Male	27 (52.9%)	9 (37.5%)	1.599	0.070
Age (years)	51 ± 8.83	49 ± 11.39	41.299	0.324
Hypertension	19 (37.5%)	3 (12.5%)	4.825	0.360
Diabetes	10 (19.6%)	4 (16.6%)	0.093	0.753
Smoking	13 (25.5%)	5 (20.8%)	0.195	0.538
Coronary disease	19 (37.2%)	7 (29.16%)	0.195	0.538
TC	4.902 ± 0.77	4.01 ± 0.73	62.745	0.003
TG	1.91 ± 1.13	1.25 ± 0.33	58.150	0.724
HDL	2.44 ± 0.84	2.38 ± 0.57	59.681	0.065
LDL	1.42 ± 0.65	1.21 ± 0.96	38.056	0.296
UA	306.11 ± 68.93	316 ± 63.05	58.15	0.772

TC: cholesterol; TG: triglycerides; HDL: high-density lipoprotein; LDL: low-density lipoprotein; UA: uric acid.

MB depth, length, and length-to-depth ratio: Based on the myocardial thickness covering the coronary arteries, patients were categorized into the Superficial MB group and the Deep MB group, as shown in Table 2. A myocardial thickness of ≥1 mm and <2 mm was classified as a superficial MB, while a thickness of ≥2 mm was classified as a deep MB. The length of the MB during both systole and diastole was significantly greater in the deep MB group compared to the superficial MB group (P < 0.001), further confirming the validity of this classification. The length of the MB in the deep MB group was 23.879 ± 10.278 mm during diastole and 23.117 ± 10.278 mm during systole, significantly longer than in the superficial MB group, which measured 16.559 ± 9.750 mm during diastole and 15.766 ± 9.122 mm during systole (Diastole: P = 0.002, Systole: P = 0.007). When comparing the length-to-depth ratio between the two groups, the deep MB group had a ratio of 6.810 ± 2.995 during systole, significantly lower than the superficial MB group’s 10.504 ± 5.849 (P = 0.010). During diastole, the Deep MB group had a ratio of 7.552 ± 3.428, also lower than the superficial MB group’s 11.311 ± 5.751 (P = 0.003), with the difference being more pronounced during diastole.

**TABLE 2:** Morphological parameters.

		Superficial MB group (n = 29)	Deep MB group (n = 22)	p value
MB depth (mm)	Diastole	1.457 ± 0.320	3.240 ± 0.898	<0.001
	Systole	1.527 ± 0.323	3.472 ± 0.890	<0.001
MB length (mm)	Diastole	16.559 ± 9.750	23.879 ± 10.278	0.002
	Systole	15.766 ± 9.122	23.117 ± 9.350	0.007
length depth ratio	Diastole	11.311 ± 5.751	7.552 ± 3.428	0.003
	Systole	10.504 ± 5.849	6.810 ± 2.995	0.010

**FIGURE 3:**
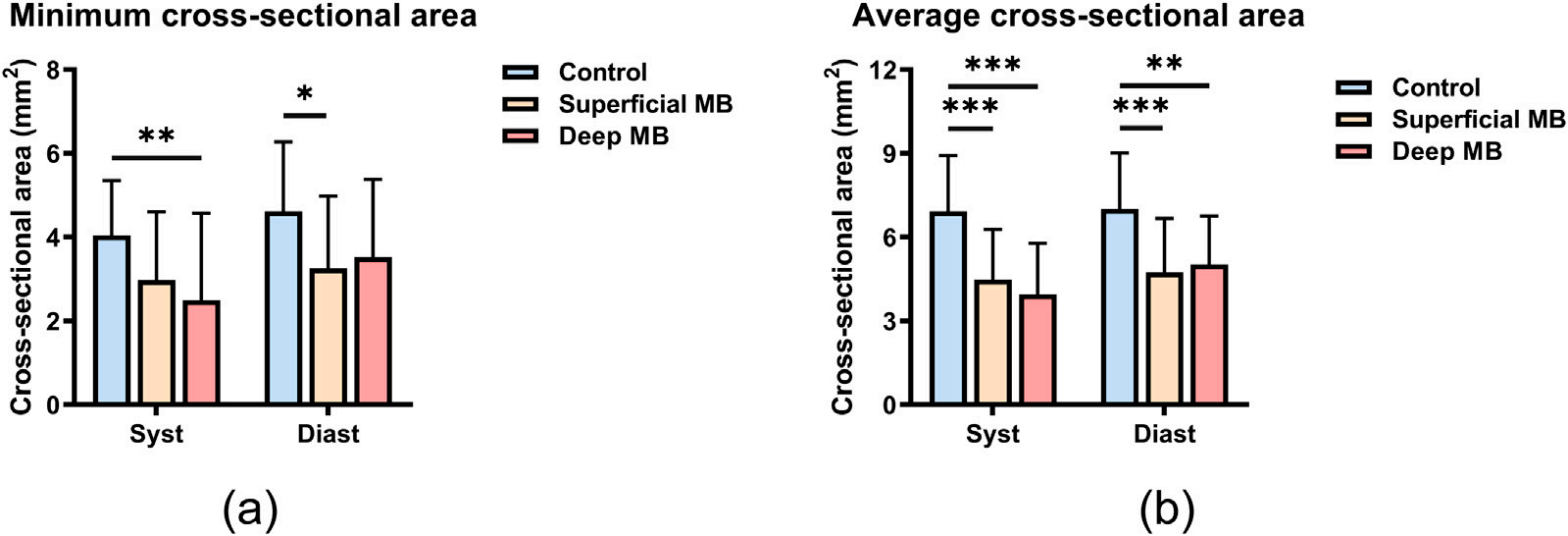
Minimum **(A)** and average **(B)** cross-sectional area at systole and diastole.

Cross-sectional area: The minimum and average cross-sectional areas among the control group, the superficial MB group, and the deep MB group, as shown in Figure 3. During systole, there was a trend of decreasing minimum cross-sectional area with increasing MB depth. However, during diastole, the minimum cross-sectional area was greater in the Deep MB group compared to the superficial MB group (as shown in Figure 3A). The minimum cross-sectional area in the control group was greater than that in the MB groups during both systole and diastole, at 4.044 ± 1.316 mm^2^ and 4.619 ± 1.659 mm^2^, respectively. There was a significant difference between the control group and the Deep MB group during systole (P = 0.008), and between the control group and the superficial MB group during diastole (P = 0.018). The average cross-sectional area of the MB segment, as shown in Figure 3B, exhibited a similar trend to the minimum cross-sectional area across both cardiac cycles, with more pronounced differences between the control group and MB groups. During systole, the average cross-sectional area in the control group was 6.927 ± 1.999 mm^2^, significantly greater than in the superficial MB group (4.486 ± 1.794 mm^2^, P < 0.001) and the deep MB group (3.956 ± 1.826 mm^2^, P < 0.001). During diastole, the average cross-sectional area in the control group was 7.014 ± 2.006 mm^2^, significantly greater than in the superficial MB group (4.737 ± 1.941 mm^2^, P < 0.001).

**FIGURE 4:**
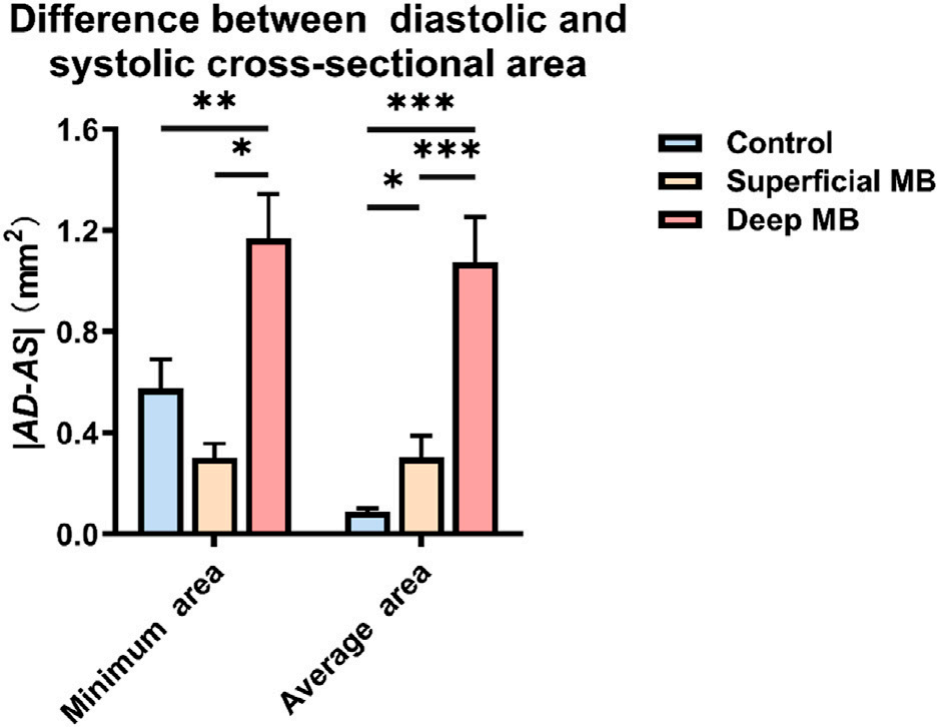
The difference between diastolic and systolic cross-sectional area.

Variations in cross-sectional area during systole and diastole: Calculate the difference in cross-sectional area between the control group, superficial MB group, and deep MB group during systole and diastole, and analyze their variations during the cardiac cycle (shown in Figure 4). Among the groups, patients with deep MB exhibited the greatest change in their minimum cross-sectional area during the cardiac cycle (1.168 ± 0.177 mm^2^), whereas those with superficial MB showed the least change (0.301 ± 0.057 mm^2^). Furthermore, significant differences were observed between the deep MB group and both the superficial MB group (P < 0.001) and the control group (0.576 ± 0.115 mm^2^, P = 0.015). However, no significant difference was found between the superficial MB group and the control group. The average cross-sectional area of the three groups demonstrated a more pronounced trend: as the depth of the muscle bridge increased, the difference in average cross-sectional area between systole and diastole widened. Notably, the deep MB group, with the largest change (1.072 ± 0.182 mm^2^), was 12 times that of control group (0.088 ± 0.014 mm^2^) and 3.6 times that of superficial MB group (0.302 ± 0.087 mm^2^). The differences among the three groups were statistically significant.

**FIGURE 5:**
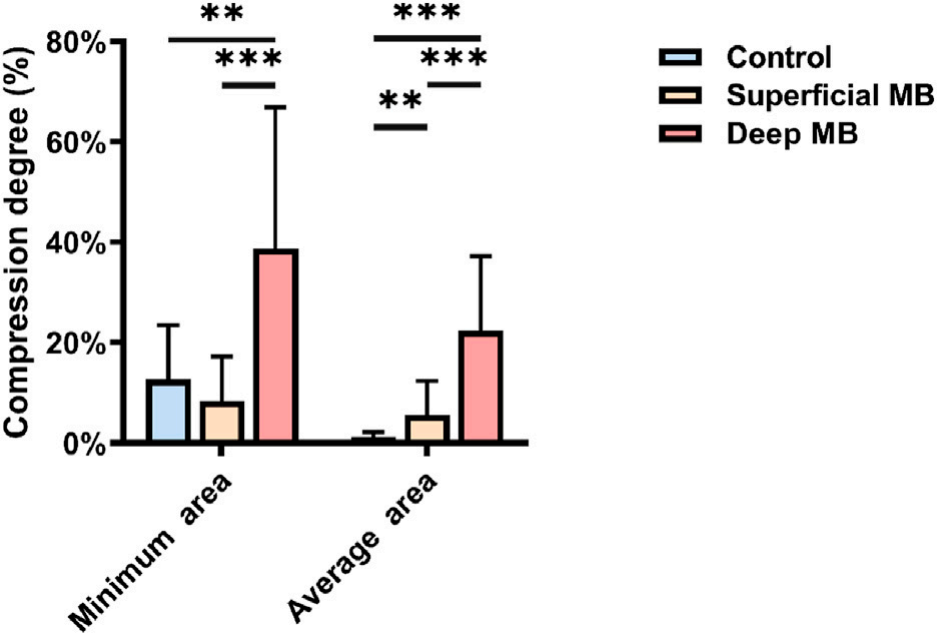
The compression degree of three group.

Compression degree of coronary artery: The compression degree of the coronary arteries during systole and diastole for the control group, the superficial MB group and the Deep MB group were calculated, including the maximum compression corresponding to the minimum cross-sectional area and the average compression corresponding to the average cross-sectional area, as shown in Figure 5. Overall, the Deep MB group exhibited the highest compression degree, with maximum and average compression degrees of 38.71% ± 28.17% and 22.35% ± 14.89%, respectively. The maximum compression degree in the deep MB group was approximately three times that of the control group (12.69% ± 10.82%) and 4.6 times that of the superficial MB group (8.36% ± 8.85%), with significant differences between the deep MB group and both the control group and the superficial MB group (P = 0.001, P < 0.001). There was no statistically significant difference between the superficial MB group and the control group. As the depth of the MB increased, the average coronary compression degree also increased. The compression degree in the deep MB group (22.35% ± 14.89%) was approximately 17 times that of the control group (1.29% ± 0.97%, P < 0.001),and about 4 times that of the superficial MB group (5.60% ± 6.78%, P < 0.001). Superficial MB group was 4.3 times of control group (P = 0.009).

**FIGURE 6:**
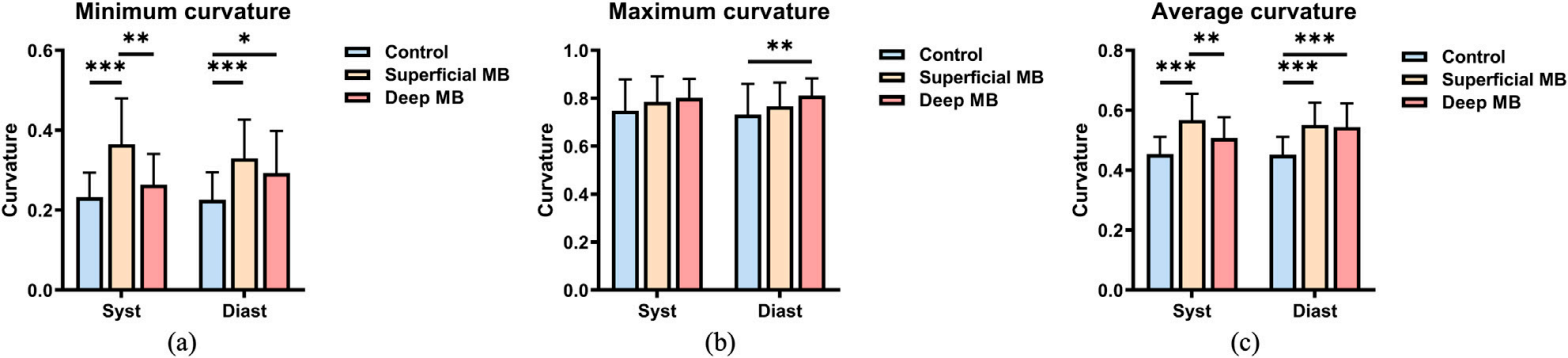
Curvature of MB, **(A)** is the minimum curvature, **(B)** is the maximum curvature, **(C)** is the average curvature.

Curvature: The minimum curvature, maximum curvature, and average curvature of the coronary arteries shown in Figure 6. Throughout cardiac pulsation, the curvature exhibited no significant changes during diastole and systole (p > 0.1). Among the three groups, there was no significant difference in maximum curvature during systole, but during diastole, the deep MB group exhibited a significantly higher maximum curvature (0.811 ± 0.073) compared to the control group (0.731 ± 0.129, P = 0.036). When comparing the minimum curvature among the three groups, both during systole and diastole, a consistent trend was observed where the superficial MB group had the highest curvature and the control group had the lowest, as illustrated in Figure 6B. During systole, there were significant differences between the superficial MB group and both the control and deep MB groups (P < 0.001, P = 0.001), while during diastole, significant differences were observed between the superficial MB group and the control group (P < 0.001), as well as between the control group and the deep MB group (P = 0.046). Comparing the average curvature among the three groups, the trend was similar to that of the minimum curvature, with the superficial MB group having the highest curvature and the control group having the lowest, as depicted in Figure 6C. During systole, the superficial MB group’s curvature (0.568 ± 0.087) was significantly higher than that of the control group (0.454 ± 0.057, P < 0.001) and the deep MB group (0.507 ± 0.070, P = 0.013). During diastole, the control group’s curvature (0.452 ± 0.059) was significantly lower than that of the superficial MB group (0.551 ± 0.075, P < 0.001) and the deep MB group (0.554 ± 0.079, P < 0.001), with no significant difference observed between the superficial MB group and the deep MB group.

**FIGURE 7:**
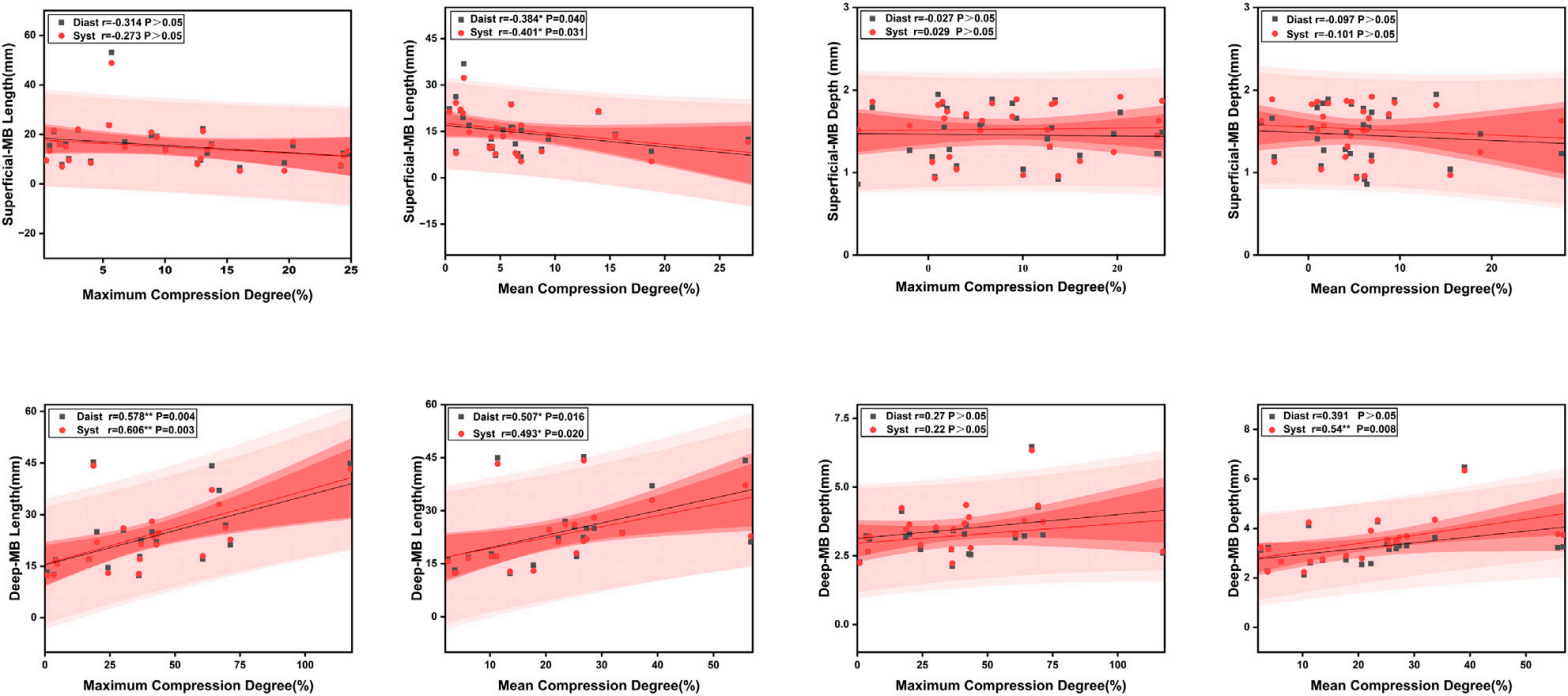
Correlation analysis: compression degree with length and depth.

Correlation analysis of parameters: Correlation analysis was conducted on the length, depth, maximum compression degree, and mean compression degree of the superficial and deep MB groups during different cardiac phases (shown in Figure 7). Within the superficial MB group, there is a mild negative correlation between the length of the muscle bridge and the mean compression length in diastole (r = −0.384, P = 0.040) and systole (r = −0.401, P = 0.031), while no correlation was observed between the depth of the superficial MB and the compression degree. For the deep MB group, the length demonstrated a moderate positive correlation with the maximum compression degree during both diastole (r = 0.578, P = 0.004) and systole (r = 0.606, P = 0.003), and a moderate positive correlation was also observed between the length of the deep MB and the mean compression degree during both diastole (r = 0.507, P = 0.016) and systole (r = 0.493, P = 0.020). Furthermore, the depth of the MB showed a moderate positive correlation with the mean compression degree during systole (r = 0.54, P = 0.008).

## Discussion

This study conducted a detailed analysis of the morphological characteristics of deep and superficial MBs using CTA imaging, leading to the following conclusions. (1) Although the MB length in the deep MB group is greater than that in the superficial MB group, the length-to-depth ratio is smaller in the deep MB group. (2) The cross-sectional area of the coronary arteries in the superficial MB group is smaller than that of normal individuals. However, as the depth of the MB increases, there is no significant difference in the coronary cross-sectional area. (3) Patients with deep MB exhibit the greatest changes in cross-sectional area during the cardiac cycle. As the depth of the MB increases, the disparity in average cross-sectional area between the systole and diastole phases widens. (4) The compression degree is greatest in the deep MB group, with the average compression degree being approximately 17 times that of normal individuals and about 4.6 times that of patients with superficial MBs. (5) The coronary curvature in patients with MB is greater than that in normal individuals, and as the depth of the MB increases, the average curvature decreases.

The morphological analysis method of the length, depth, maximum compression degree, and average compression degree of superficial and deep muscle bridge groups at different cardiac phases (diastole and systole) is not entirely consistent with previous literature. In clinical practice, the relationship between length, depth, and compression degree remains controversial (Sternheim et al., 2021; Zhang et al., 2024). The relationship between compression degree and spasm was found that MB length is positively correlated with the severity of compression and significantly associated with spasm symptoms (Saito et al., 2017). This may be due to endothelial dysfunction caused by the long-term compression-relaxation state of MB, which enhances vascular reactivity. In contrast, Arjmand Shabestari et al. (2016) suggested that compression level is only weakly correlated with length but strongly correlated with depth. This could be because superficial myocardial fibers longitudinally cover the coronary arteries, while deeper MB myocardial fibers can encircle the vessels (Zhu et al., 2020). The hemodynamics of MB might be influenced by the direction of myocardial fibers covering the coronary arteries; therefore, MB depth is more likely to trigger clinical symptoms (Uusitalo et al., 2016).

In our study, the compression degree showed mostly mild or no statistical significance in relation to the length of the MB, whereas there was a moderate to strong correlation between compression and depth. Uusitalo found that superficial myocardial fibers only longitudinally cover the coronary veins, while deep MB fibers can encircle the vessels (Uusitalo et al., 2016). The hemodynamic effects of the bridge may be influenced by the orientation of the myocardial fibers covering the coronary arteries. Consequently, the depth of the MB is more likely to lead to clinical manifestations. Kim used dual-source CT and MB morphology, the correlation between systolic lumen narrowing and mid-segment LAD MB length was poor, whereas the correlation between length and MB depth was moderate (Kim et al., 2011). Previous studies have shown controversy in predicting the characteristics of dynamic compression, as changes in anatomical properties were primarily focused on accompanying atherosclerosis (Zhang et al., 2021; Matta et al., 2024).

There are two potential mechanisms by which myocardial bridging affects hemodynamics (Okada et al., 2020). The first is diastolic filling dysfunction, where the compression of the vessel by the MB during systole persists into the mid-to-late diastole, leading to hemodynamic disturbances and filling dysfunction during both systole and diastole (Corban et al., 2014). The second mechanism involves myocardial compression causing proximal vessel constriction, resulting in restricted blood flow downstream (Herrmann et al., 2004). In this study, the minimum and average cross-sectional areas differed among deep MB, superficial MB, and non-MB cases. However, the minimum cross-sectional area is more variable and less reliable than the average cross-sectional area in accurately reflecting the true situation.

### Limitations

First, this study did not use coronary angiography as a control. Additionally, distal MB patients were not included. Although some reports suggest that distal MBs account for two-thirds of all MBs, they do not cause systolic coronary artery stenosis or have corresponding clinical symptoms (Hwang et al., 2010).

## Conclusion

This study examines the morphological differences among normal individuals, those with superficial MB, and those with deep MB under different conditions of myocardial coronary coupling. A coronary artery reconstruction method based on CTA imaging was established to capture the morphological biomechanical patterns at different time points. The study highlights a stronger correlation between MB depth and compression degree, emphasizing the need for increased clinical intervention and attention towards deep MBs. Early diagnosis of MB patients holds significant importance in assessing the risk of cardiovascular emergencies.

## Data Availability

The datasets presented in this article are not readily available because Retrospective analysis of CTA data. Requests to access the datasets should be directed to zhaodl@pku.edu.cn.
